# Work ability of military police officers

**DOI:** 10.11606/s1518-8787.2019053001014

**Published:** 2019-09-18

**Authors:** Carla Requião Barreto, Liliane Lins-Kusterer, Fernando Martins Carvalho

**Affiliations:** I Universidade Federal da Bahia. Faculdade de Medicina da Bahia. Programa de Pós-Graduação em Saúde, Ambiente e Trabalho. Salvador, BA, Brasil

**Keywords:** Police, Safety, Work Capacity Evaluation, Working Conditions, Cross-Sectional Studies

## Abstract

**OBJECTIVE:**

To determine the prevalence of work ability (WA) and describe characteristics of the subgroup with poor WA among military police officers.

**METHODS:**

A descriptive and cross-sectional study with 329 male military police officers engaged in street patrolling in Salvador, Bahia, Brazil, selected by proportionate stratified sampling. The Work Ability Index and a structured form were used to collect information about age, education, marital status, housing, salary, car ownership, work hours, rank (official or enlisted), drinking, smoking, frequency of vigorous physical activity, and obesity. Data were analyzed by uni and bivariate statistical techniques.

**RESULTS:**

The work ability of the 329 military police officers was classified as poor (10.3%), moderate (28.9%), good (34.7%), and excellent (26.1%), with mean score of 37.8 and standard deviation of 7.3 points. Policemen with poor work ability, compared with those with moderate, good or excellent WA, presented higher proportions of individuals who did not own their residences (p < 0.001), with work hours above eight hours/day (p < 0.026), and obesity (p < 0.001). In the subgroup of the 26 policemen who concomitantly did not own their residences, worked more than eight 8 hours/day and were obese, the prevalence of poor work ability was 31.0%. The prevalence of poor WA was 31.0% among the 29 policemen who were simultaneously obese and did not own their residences and of 27.9% among the 43 policemen who were obese and work hours above eight hours/day.

**CONCLUSIONS:**

A high percentage of military police officers from Salvador presented poor or moderate work ability, which may hamper or compromise their policing activities. The prevalence of poor work ability was higher among the policemen who did not own their residences, worked more than 8 hours/day and were obese.

## INTRODUCTION

During work, the military police officer deals with daily stressing factors, both in the police organization environment and on the streets, facing situations of violence that generate high physical^1 , 2^ and mental^3 - 5^ demand, affecting their health and work ability^3 , 6^ . In South Korea, a cohort study followed 92,545 police officers from 2002 to 2014 and found higher incidence rates for angina pectoris, acute myocardial infarction and cerebrovascular disease than among 405,463 federal and state civil servants^7^ . Ostensive policing, the target activity of the military police organization, is an essentially dynamic and risky task originated from the common need of community safety and leads the police officer to deal with various dangerous and stressful situations^8 , 9^ . During street patrolling, this professional acts in the prevention of incidents, such as petty theft, drug trafficking, crimes against property or violent crimes^10^ .

The working conditions of military police officers in Brazil are unfavorable, considering the constant exposure to risk situations, long working hours, insufficient human resources, high levels of mental suffering, inadequate instruments, lack of preventive maintenance in equipment such as weapons, uniforms, vests and vehicles, low wages, and lack of technical training^5 , 6 , 8 , 9^ . The deleterious influences of work on the health of the military police officer are mainly related to the decrease in leisure and family time, declination of economic power, restriction of access to essential goods and services, exposure to stress, and sedentarism^11^ .

In Brazil, the few epidemiological studies on the ability of military police officers assessed convenience and small-sized samples^3 , 12^ . It is important to assess the prevalence of work ability according to its different subgroups and to describe the subgroup characteristics with poor work ability in military police officers. Such epidemiological information may support the implementation of measures to improve working and health conditions.

This study aimed to determine work ability prevalence (WA) and to describe characteristics of the subgroup with poor work ability in military police officers in the city of Salvador, Brazil.

## METHODS

A cross-sectional study was carried out with military police officers from the state of Bahia. Only male police officers executing ostensive policing activities were included. Those who were on sick leave, conducted administrative activity or were in special situations that kept them away from the corporation target activity were excluded. Samples were randomly stratified and proportional to the 27 independent companies of the military police of the city of Salvador, Bahia. This investigation is part of a larger study that also addressed the health-related quality of life of military police officers using the 36-Item Short Form Health Survey (SF-36). For this reason, sample calculation was based on the largest sample size, considering the dispersion of the quality of life construct. To calculate the sample size referring to the work ability index (WAI), formulas and procedures similar to those in the SF-36 were used. To estimate the *s* parameter, a value of 4.08 was assumed, based on a study^13^ with firemen from the city of São Paulo. The sample was calculated assuming the alpha level of 5%, power of 80% and precision of one percentage point (ability to detect a difference of one percentage point in relation to the average of reference population), which was constituted in the equation n = 7.9*(4.08)^2^ /(1)^2^ , resulting in 131 individuals. However, quality of life, outcome sought in the larger study, required a sample size n = 289, inflated by 20% to predict losses during police recruitment, resulting in a desired final sample of 347 individuals.

The work ability is conceptualized as “how good a worker is or will be at present or in the near future, and how able he or she is to perform their work according to the requirements, their state of health, and physical and mental capacities”^14^ . It involves a dynamic process that results from the interaction of the individual’s resources with physical, mental and social demands of work, work environment, organizational culture and management^15^ . The work ability index was applied^16^ , which portrays the worker’s own concept of his work ability. WAI was translated into Portuguese^16^ and validated in Brazilian populations, showing satisfactory psychometric properties regarding construct validity, criterion, reliability^17^ and test-retest reliability^18^ . Its results can be used at individual and collective levels to identify workers with impaired functional capacity or to outline the capacity profile in a group of workers, respectively^19^ . WAI consists of seven dimensions: current work ability as opposed to lifetime best, work ability in relation to the demands of the job, number of diseases diagnosed by a physician, estimated work impairment due to illness, sick leave, self-prognosis of work ability, and mental resources. Scores of each dimension were weighted according to specific characteristics of the work performed (physical or mental) and added to determine the result, which can vary from 7 to 49 points. The score is classified into four categories of work ability: poor (7 to 27 points), moderate (28 to 36 points), good (37 to 43 points), and excellent (44 to 49 points). Excellent must be maintained, good must be supported, moderate should be improved and poor should be restored, according to the proposal of the WAI creators^16^ .

A structured questionnaire collected data on age, schooling, marital status, home ownership (as their own or not, that is, rented, financed or otherwise), salary, car ownership, work hours, rank (official or enlisted), frequency of alcohol and tobacco consumption, frequency of vigorous physical activity and obesity. Obesity was measured by body mass index (BMI = weight in kilograms divided by height in square meters) from values reported by the individuals interviewed, classified as underweight (< 18.5), normal (18.5 to 24.9), overweight (25.0 to 29.9), obesity class I (30.0 to 34.9), obesity class II (35.0 to 39.9), and obesity class III (≥ 40.0)^22^ . For the purpose of the statistical analyses of this study, a police officer with BMI ≥ 30.0 was considered obese.

Descriptive analyses were performed using SPSS software *,* version 20.0 (IBM Corp., Armonk, NY, USA). In bivariate analyses for statistical inferences of differences among proportions, the chi-square test was applied and, when indicated, the Fisher test, using the OpenEpi software version 3.01^a^ .

All randomly selected policemen participated voluntarily in the survey. After explaining the objectives, participants signed a free informed consent form. All information related to the participants was confidential. The study was approved by the Ethics Committee in Research on Human Beings of the Medical School of Bahia for opinion number 554,724.

## RESULTS

Of 347 military officers selected for the study, 18 were excluded because the information of the WAI questionnaire was incomplete. The WAI mean score of the remaining 329 police officers was 37.8, with a standard deviation of 7.3, ranging from 14 to 49 points. In this group, 10.3% had poor work ability, 28.9% moderate, 34.7% good, and 26.1% excellent. The subgroup with poor WA was distinguished from the others by its low prevalence and a relatively narrow 95%CI ( Table 1 ).

**Table 1:** Prevalence (%) and respective confidence intervals of 95% (95%CI) of work ability in four subgroups of 329 military police officers. Salvador, BA, 2014.

Work ability	n	%	95%CI
Excellent	86	26.1	16.8–35.4
Good	114	34.7	26.043.4
Moderate	95	28.9	19.8–38.0
Poor	34	10.3	7.0–3.6

Total	329	100.0	

Compared with policemen with moderate, good or excellent WA, the subgroup with poor WA had significantly higher proportions of individuals who did not own their residences (21.1% *versus* 3.52%, p < 0.001), work hours higher than eight hours (11.7% *versus* 3.0%, p < 0.026), and obesity (25.5% *versus* 7.8%, p < 0.001). Among the 47 obese policemen, 25.5% individuals had poor work ability, while among the 282 non-obese policemen, this prevalence was only 7.8%. ( Table 2 ).

**Table 2:** Work ability according to characteristics of military police officers. Salvador, BA, 2014.

Characteristics	N	Work ability				p*

		Poor		Moderate, good or excelent		
	
		n	%	n	%	
Age group (years)						
24-44	276	27	9.8	249	90.2	0.453.
45-54	53	7	13.2	46	86.8	
Schooling						
High School	212	18	8.5	194	915	0.139
Higher education/Graduation	117	16	13.7	101	86.3	
Marital status						
Married/stable relationship	201	24	11.9	177	88.1	0.332
Single/separate/widowed	128	10	7.8	118	92.2	
Home ownership						
Yes	201	7	3.5	194	96.5	
No	128	27	21.1	101	78.9	0.001
Rank						
Officer	25	1	4.0	24	96.0	0.502
Enlisted	300	32	10.7	268	89.3	
Salary						
2 to 6 minimum wages	298	32	10.7	266	89.3	0.706
> 6 minimum wages	31	2	6.5	29	93.5	
Car ownership						
No	98	15	15.3	83	84.5	0.058
Yes	228	19	8.3	209	91.7	
Working hours						
≤ 8h/day	67	2	3.0	65	97.0	0.026
> 8h/day	262	32	11.7	230	88.3	
Alcohol						
Do not consume/social consumption	305	29	9.5	276	90.5	0.079
Frequent consumption	24	5	20.8	19	79.2	
Smoking						
Non-smoker/former smoker	310	31	10.0	279	90.0	
Smoker	19	3	15.8	16	84.2	
Vigorous physical activity						
0–2 days per week	157	18	11.5	139	88.6	
3–7 days per week	172	16	9.3	156	90.7	0.519
Obesity (BMI ≥ 30.0)						
No		22	7.8	260	92.2	0.001
Yes	47	12	25.5	35	74.5	

BMI: body mass index.

* Chi-square test and, when indicated, Fisher’s test.

In the group of 26 policemen who concomitantly did not own their residences, worked more than eight hours a day and were obese, the prevalence of poor work ability was 31.0% ( Table 3 ). This prevalence was 31.0% in the 29 policemen who were simultaneously obese and did not own their residences, and 27.9% in the 43 policemen who were simultaneously obese and had an eight-hour workday ( Table 4 ).

**Table 3:** Prevalence of poor work ability (WA) according to combinations of the substrates workday, housing and obesity in 329 military police officers. Salvador, BA, 2014.

Workday > 8h/day	Non-owned residence*	Obese	N	Prevalence of poor WA	

				n	%
Yes	Yes	Yes	26	9	31.0
Yes	No	Yes	17	3	17.6
Yes	Yes	No	148	17	11.5
Yes	No	No	71	3	4.2
No	Yes	Yes	3	0	0.0
No	No	Yes	1	0	0.0
No	Yes	No	44	1	2.3
No	No	No	19	1	5.3
-	-	-	329	34	10.3

* Rented, financed or other situations

**Table 4:** Prevalence of poor work ability (WA) according to combinations of the substrates workday, housing and obesity, taken two to two, in 329 military police officers. Salvador, BA, 2014.

Workday > 8h/day	Non-owned residence*	Obese	N	Prevalence of poor WA	

				n	%
-	Yes	Yes	29	9	31.0
-	Yes	Yes	198	18	9.4
-	No	No	18	3	16.7
-	No	No	90	4	4.4
-	-	-	329	34	10.3
Yes	-	Yes	43	12	27.9
Yes	-	Yes	4	0	0.0
No	-	No	219	20	9.1
No	-	No	63	2	3.2
-	-	-	329	34	10.3
Yes	Yes	-	174	26	14.9
Yes	Yes	-	47	1	2.1
No	No	-	88	6	6.8
No	No	-	20	1	5.0
-	-	-	329	34	10.3

* Rented, financed or other situations

## DISCUSSION

The 329 military police officers from Salvador had a WAI mean score of 37.8 points, which would put this population in the category of good WA. Among those 329 policemen, 95 (28.9%) with moderate WAI should have their work ability improved, and 34 (10.3%) with poor WAI should have their work ability restored, according to classification proposed by the instrument creators^23^ .

The work ability of military police officers from Salvador was similar to that reported for 98 military police officers (86 men) from Uberaba, Minas Gerais: 22.4% of workers with excellent ability, 37.8% with good ability, 29.6% with moderate ability, and 10.2% with poor ability^3^ . This parameter was also assessed in 42 police officers of the Special Operations Battalion of Santa Maria, in Rio Grande do Sul. This battalion is prepared to act in special situations, such as bank robberies, kidnappings and riots, and its members must present excellent physical and mental conditioning. In fact, officers in the Special Operations Battalion of Santa Maria were characterized by a high proportion of individuals with excellent (40.5%) and good (47.6%) work ability and a lower proportion in the moderate (11.9%) and poor (0.0%) WA strata^12^ . In 94 Finnish police officers, the average WAI was 39 points, poor in 7% of the sample, moderate in 21%, good in 57%, and excellent in 72% of the sample^2^ . These proportions show a better work ability than those found in police officers from Salvador, with the exception that the average age of the Finnish police was way higher (49 years, ranging from 42 to 61) than that of policemen from Salvador (36.5 years, ranging from 25 to 54).

Some studies have evaluated the work ability in other occupational groups. A study carried out with 30 military firefighters from a city in the state of São Paulo did not find individuals in the strata of poor work ability; the others were classified in the strata of excellent (53.3%), good (36.7%) and moderate (10.0%) work ability^13^ .

In summary, considering the results of the five studies mentioned, the military police from Salvador are characterized by a high proportion of individuals in poor and moderate work ability strata, in proportions comparable only with military police officers from Uberaba^3^ . The subgroup with poor work ability was characterized by a high proportion of police officers who worked more than eight hours a day, did not own their residences and were obese.

Long working hours have been associated with a decrease in work ability, due to stress provoked by physical effort, in metal workers and retail trade workers^20^ . Physical effort is a well-established risk factor for work ability^21^ .

Studies reporting association between poor work ability and home ownership are beyond our knowledge. Not owning a house (rented, financed or other forms of property possession) must be taken as a social condition proxy of the military police officer. In this case, not owning a house would be a link in the association between low social status and poor work ability.

Our results revealed the importance of obesity in the poor work ability of police officers. The prevalence of obesity was much higher in military police with poor WA (25.5%) than in police with moderate, good and excellent WA (7.8%). In addition, analysis of the combinations of housing, workday and obesity substrates showed how much the last variable contributed to poor WA prevalence of the investigated police officers. Being overweight represents a relevant problem for the work ability of these police officers, whose ostensible policing activity in public environments usually require great physical demand. Obesity is associated with increased risks of heart diseases, high blood pressure, stroke, certain types of cancer, diabetes, biliary lithiasis, dyslipidemia, osteoarthritis, gout, lung diseases and sleep apnea, as well as prejudice and discrimination^22^ . Surveys on quality of life shows that obesity is more associated with the worsening of physical than emotional aspects and with the worsening of physical capacity, vitality and body pain^23^ .

The nature of exposures and the outcome that were objects of this descriptive survey, does not allow us to be sure about the assumptions for causality investigation, such as: a) that the exposure preceded the outcome in the time line; b) non-existence of reverse causality; c) existence of a stable population over a given period of time; and d) the mean duration of the outcome should be the same regardless of the exposure group^24 , 25^ . Moreover, one cannot dismiss the importance of the “healthy worker effect”^26^ , which may have acted in a more relevant way in the composition of this group of military police officers in at least two ways: a) by entering the corporation and selecting individuals with different characteristics to make up the exposure subgroups of and outcome and b) over time in the corporation, excluding those police officers who were presenting poor work ability for different reasons (survival effect of the healthy worker). An important limitation of this investigation was the lack of assessment of work organization, workload and psychosocial workload. The inherent limitations of a cross-sectional study was added to the difficulty of working with a vulnerable population. It was necessary to keep the respondents anonymous to minimize influences of characteristic hierarchical relations in military organizations. During data collection, survey was widely publicized to sensitize police officers to participate, which enables a program implementation aimed at improving their health-related quality of life. In addition to the aspects mentioned, the collection by a single health professional, belonging to the corporation, enabled the unusual proportion of formal refusals found in this study (0.0%). A strong point of this study was the use of a randomly selected sample, relatively large, representative of military police officers from Salvador.

## CONCLUSIONS

One in 10 military police officers from Salvador has poor work ability, which may hamper or compromise the performance of their ostensive policing activities. This subgroup of professionals is characterized by a high proportion of individuals who did not own a house, worked more than eight hours a day and were obese.

## Footnotes

a Dean AG, Sullivan KM, Soe MM. OpenEpi: open source epidemiologic statistics for Public Health [computer program]. Version 3.01, updated 2013 [cited 2018 Sep 2]. Available from: https://www.openepi.com/
