# Supplementary material for: Work ability of military police officers
Source: Rev Saude Publica. 2019 Sep 18;53:79. doi: 10.11606/s1518-8787.2019053001014 (PMC6763285; doi:10.11606/s1518-8787.2019053001014)
Supplement: Translation

# Translation: Capacidade para o trabalho de policiais militares

**Authors:** Carla Requião Barreto, Liliane Lins-Kusterer, Fernando Martins Carvalho

**Affiliations:** I Universidade Federal da Bahia. Faculdade de Medicina da Bahia. Programa de Pós-Graduação em Saúde, Ambiente e Trabalho. Salvador, BA, Brasil

**Keywords:** Polícia, Segurança, Avaliação da Capacidade de Trabalho, Condições de Trabalho, Estudos Transversais

## Abstract

**OBJETIVO:**

Determinar a prevalência da capacidade para o trabalho (CT) e descrever características do subgrupo com baixa CT em policiais militares.

**MÉTODOS:**

Estudo descritivo, de corte transversal, investigando 329 policiais militares do sexo masculino que faziam policiamento ostensivo em Salvador, BA, Brasil, selecionados por amostragem estratificada com partilha proporcional. Utilizaram-se o índice de capacidade para o trabalho e um questionário estruturado para coletar informações sobre idade, escolaridade, estado civil, moradia, salário, posse de carro, jornada de trabalho, classe (oficial ou praça), consumo frequente de álcool e tabaco, frequência de atividade física vigorosa e obesidade. Os dados foram analisados com o uso de técnicas estatísticas uni e bivariadas.

**RESULTADOS:**

A capacidade para o trabalho dos 329 policiais foi classificada como baixa (10,3%), moderada (28,9%), boa (34,7%) ou ótima (26,1%), com escore médio de 37,8 e desvio-padrão de 7,3 pontos. Policiais com baixa capacidade para o trabalho, comparados àqueles com CT moderada, boa ou ótima, apresentaram maiores proporções de indivíduos com moradia não própria (p < 0,001), com jornada de trabalho maior que oito horas por dia (p < 0,026) e obesos (p < 0,001). No subgrupo dos 26 policiais que concomitantemente residiam em moradia não própria, tinham jornada de trabalho maior que oito horas por dia e eram obesos, a prevalência de baixa CT foi de 31,0%. A prevalência de baixa CT foi de 31,0% nos 29 policiais que, simultaneamente, eram obesos e residiam em moradia não própria e de 27,9% nos 43 policiais obesos com jornada de trabalho maior que oito horas por dia.

**CONCLUSÕES:**

Elevada proporção dos policiais militares de Salvador apresentou baixa ou moderada capacidade para o trabalho, o que pode dificultar ou comprometer o desempenho de sua ocupação. A prevalência de baixa capacidade para o trabalho foi maior em policiais que residiam em moradia não própria, trabalhavam mais que oito horas por dia e, principalmente, eram obesos.

## INTRODUÇÃO

No seu trabalho, o policial militar convive cotidianamente com fatores estressores, tanto no ambiente das organizações policiais militares como nas ruas, enfrentando situações de violência que geram alta demanda física^1 , 2^ e mental^3^ e podem afetar fortemente sua saúde e capacidade para o trabalho^3 , 6^ . Na Coreia do Sul, um estudo de coorte acompanhou 92.545 policiais de 2002 a 2014 e constatou taxas de incidência mais elevadas para angina pectoris, infarto agudo do miocárdio e doença cerebrovascular do que entre 405.463 funcionários públicos federais e estaduais^7^ . O policiamento ostensivo, atividade-fim da organização policial militar, é uma tarefa essencialmente dinâmica e arriscada que tem origem na necessidade comum de segurança da comunidade e leva o policial a lidar com diversas situações perigosas e estressantes^8 , 9^ . Durante o patrulhamento nas ruas, esse profissional atua na prevenção de ocorrências como pequenos furtos, tráfico de drogas, crimes contra o patrimônio ou crimes violentos letais intencionais^10^ .

As condições de trabalho dos policiais militares no Brasil são desfavoráveis, considerando a exposição constante a situações de risco, longas jornadas de trabalho, efetivo humano insuficiente, elevado nível de sofrimento mental, instrumentos inadequados, falta de manutenção preventiva em equipamentos como armas, fardas, coletes e viaturas, baixos salários e falta de capacitação técnica^5 , 6 , 8 , 9^ . As influências deletérias do trabalho na saúde do policial militar estão relacionadas, principalmente, à diminuição do tempo dedicado ao lazer e à família, à deterioração do poder econômico, à restrição de acesso a bens e serviços essenciais, à exposição a agentes estressores e ao sedentarismo^11^ .

No Brasil, os poucos estudos epidemiológicos realizados sobre a capacidade para o trabalho de policiais militares avaliaram amostras de conveniência e de tamanho reduzido^3 , 12^ . É importante avaliar a prevalência de capacidade para o trabalho segundo seus diferentes subgrupos e descrever as características do subgrupo com baixa capacidade para o trabalho em policiais militares. Essas informações epidemiológicas podem fundamentar a implementação de medidas para a melhoria das condições de trabalho e saúde desses profissionais.

Este estudo objetivou determinar a prevalência da capacidade para o trabalho (CT) e descrever características do subgrupo com baixa capacidade para o trabalho em policiais militares da cidade de Salvador, Brasil.

## MÉTODOS

Realizou-se um estudo de corte transversal com policiais militares do estado da Bahia. Foram incluídos apenas policiais do sexo masculino que desempenhavam atividade de policiamento ostensivo. Foram excluídos aqueles que estavam em licença médica, que exerciam atividade administrativa ou que estavam em situações especiais que os afastavam da atividade-fim da corporação. Procedeu-se a uma amostragem aleatória estratificada e proporcional às 27 companhias independentes da polícia militar da cidade de Salvador, Bahia. Este trabalho faz parte de um estudo maior em que também foi abordada a qualidade de vida relacionada a saúde de policiais militares, usando o questionário *36-Item Short Form Health Survey* (SF-36). Por esse motivo, o cálculo amostral baseou-se no maior tamanho da amostra, considerando a dispersão do construto qualidade de vida. Para o cálculo do tamanho amostral referente ao índice de capacidade para o trabalho (ICT), utilizaram-se fórmulas e procedimentos semelhantes aos utilizados no SF-36. Para estimativa do parâmetro *s* , assumiu-se o valor de 4,08, a partir de um estudo^13^ com bombeiros da cidade de São Paulo. A amostra foi calculada assumindo o nível alfa de 5%, poder de 80% e precisão de um ponto percentual (capacidade de detectar uma diferença de um ponto percentual em relação à média da população de referência), o que se constituiu na equação n = 7,9*(4,08) ^2^ /(1)^2^, resultando em 131 indivíduos. No entanto, o desfecho qualidade de vida pesquisado no estudo maior precisou de amostra de tamanho n = 289, inflada em mais 20% para prever perdas durante o recrutamento de policiais, resultando numa amostra final desejada de 347 indivíduos.

A capacidade para o trabalho é conceituada como “quão bem está, ou estará, um(a) trabalhador(a) presentemente ou num futuro próximo, e quão capaz ele ou ela pode executar seu trabalho em função das exigências, de seu estado de saúde e capacidades físicas e mentais”^14^ . Ela envolve um processo dinâmico que resulta da interação dos recursos do indivíduo com as demandas físicas, mentais e sociais do trabalho, ambiente de trabalho, cultura organizacional e seu gerenciamento^15^ . Foi aplicado o instrumento índice de capacidade para o trabalho^16^ , que retrata o próprio conceito do trabalhador sobre sua capacidade para o trabalho. O ICT foi traduzido para o português^16^ e validado em populações brasileiras, mostrando propriedades psicométricas satisfatórias quanto à validade de construto, de critério, confiabilidade^17^ e confiabilidade teste-reteste^18^ . Seus resultados podem ser utilizados nos níveis individual e coletivo, para identificar trabalhadores com comprometimento da capacidade funcional ou para traçar o perfil da capacidade para o trabalho de um grupo de trabalhadores, respectivamente^19^ . O ICT é constituído por sete dimensões: capacidade para o trabalho atual comparada com a melhor de toda a vida, capacidade para o trabalho em relação às exigências do trabalho, número de doenças diagnosticadas por médico, perda estimada para o trabalho devido a doenças, faltas ao trabalho por doenças, prognóstico próprio sobre a capacidade para o trabalho e recursos mentais. As pontuações de cada dimensão foram ponderadas conforme as características específicas da atividade realizada no trabalho (físicas ou mentais) e adicionadas para determinar o resultado final, que pode variar de 7 a 49 pontos. A pontuação é classificada em quatro categorias de capacidade para o trabalho: baixa (7 a 27 pontos), moderada (28 a 36 pontos), boa (37 a 43 pontos) e ótima (44 a 49 pontos). A ótima deve ser mantida, a boa deve ser apoiada, a moderada deve ser melhorada e a baixa deve ser restaurada, segundo proposta dos criadores do ICT^16^ .

Num questionário estruturado foram coletados dados sobre idade, escolaridade, estado civil, posse da moradia (estratificada em própria ou não própria, ou seja, alugada, financiada ou outras), salário, posse de carro, jornada de trabalho, classe (oficial ou praça), consumo frequente de álcool e tabaco, frequência de atividade física vigorosa e obesidade. A obesidade foi medida por meio do índice de massa corporal (IMC = peso em quilos dividido pela altura em metros ao quadrado) a partir de valores referidos pelos indivíduos entrevistados, classificado como abaixo do peso (<18,5), normal (18,5 a 24,9), pré-obeso (25,0 a 29,9), obesidade grau I (30,0 a 34,9), obesidade grau II (35,0 a 39,9) e obesidade grau III (≥ 40,0)^22^ . Para efeito das análises estatísticas deste trabalho, considerou-se obeso o policial com IMC ≥ 30,0.

As análises descritivas foram realizadas com uso do software SPSS *,* versão 20.0 (IBM Corp., Armonk, NY, USA). Nas análises bivariadas para inferências estatísticas de diferenças entre proporções, foi utilizado o teste qui-quadrado e, quando indicado, o teste de Fisher, com auxílio do software OpenEpi versão 3.01^a^ .

Todos os policiais sorteados consentiram em participar voluntariamente da pesquisa. Após explicação dos objetivos, os participantes assinaram um termo de consentimento livre e esclarecido. Todas as informações relacionadas aos participantes foram confidenciais. A pesquisa foi aprovada pelo Comitê de Ética em Pesquisa em Seres Humanos da Faculdade de Medicina da Bahia pelo parecer número 554.724.

## RESULTADOS

Dos 347 policiais militares selecionados para o estudo, 18 foram excluídos porque as informações do questionário do ICT estavam incompletas. O escore médio do ICT dos 329 policiais restantes foi 37,8, com desvio-padrão de 7,3, variando de 14 a 49 pontos. Nesse grupo, 10,3% apresentaram capacidade para o trabalho baixa, 28,9% moderada, 34,7% boa e 26,1% ótima. O subgrupo com baixa CT distinguiu-se dos demais por sua baixa prevalência e IC95% relativamente estreito ( Tabela 1 ).

**Tabela 1:** Prevalência (%) e respectivos intervalos de confiança de 95% (IC95%) de capacidade para o trabalho em quatro subgrupos de 329 policiais militares. Salvador, BA, 2014.

Capacidade para o trabalho	n	%	IC95%
Ótima	86	26,1	16,8–35,4
Boa	114	34,7	26,0–43,4
Moderada	95	28,9	19,8–38,0
Baixa	34	10,3	7,0–13,6

Total	329	100,0	-

Comparado aos policiais com CT moderada, boa ou ótima, o subgrupo de policiais com CT baixa apresentou proporções significantemente maiores de indivíduos com moradia não própria (21,1% *versus* 3,52%; p < 0,001), jornada de trabalho maior que oito horas por dia (11,7% *versus* 3,0%; p < 0,026) e obesidade (25,5% *versus* 7,8%; p < 0,001). Entre os 47 policiais obesos, havia 25,5% de indivíduos com baixa capacidade para o trabalho, enquanto entre os 282 não obesos essa prevalência era de apenas 7,8%. ( Tabela 2 ).

**Tabela 2:** Capacidade para o trabalho segundo características de policiais militares. Salvador, BA, 2014.

Característica	N	Capacidade para o trabalho				p*

		Baixa		Moderada, boa ou ótima		
	
		n	%	n	%	
Faixa etária (anos)						
24–44	276	27	9,8	249	90,2	0,453
45–54	53	7	13,2	46	86,8	
Escolaridade						
Ensino médio	212	18	8,5	194	91,5	0,139
Superior/pós-graduação	117	16	13,7	101	86,3	
Situação conjugal						
Casado/relação estável	201	24	11,9	177	88,1	0,332
Solteiro/separado/viúvo	128	10	7,8	118	92,2	
Moradia própria						
Sim	201	7	3,5	194	96,5	
Não	128	27	21,1	101	78,9	0,001
Classe						
Oficial	25	1	4,0	24	96,0	0,502
Praça	300	32	10,7	268	89,3	
Salário						
2 a 6 salários mínimos	298	32	10,7	266	89,3	0,706
> 6 salários mínimos	31	2	6,5	29	93,5	
Tem carro						
Não	98	15	15,3	83	84,5	0,058
Sim	228	19	8,3	209	91,7	
Jornada de trabalho						
≤ 8 h/dia	67	2	3,0	65	97,0	0,026
> 8 h/dia	262	32	11,7	230	88,3	
Álcool						
Não consome/consumo social	305	29	9,5	276	90,5	0,079
Consumo frequente	24	5	20,8	19	79,2	
Fumo						
Não fumante/ex-fumante	310	31	10,0	279	90,0	0,421
Fumante	19	3	15,8	16	84,2	
Atividade física vigorosa						
0–2 dias/semana	157	18	11,5	139	88,6	0,519
3–7 dias/semana	172	16	9,3	156	90,7	
Obeso (IMC ≥ 30,0)						
Não	282	22	7,8	260	92,2	0,001
Sim	47	12	25,5	35	74,5	

IMC: índice de massa corporal

* Teste qui-quadrado e, quando indicado, teste de Fisher.

No conjunto dos 26 policiais que concomitantemente residiam em moradia não própria, tinham jornada de trabalho maior que oito horas por dia e eram obesos, a prevalência de baixa capacidade para o trabalho foi de 31,0% ( Tabela 3 ). Essa prevalência foi de 31,0% nos 29 policiais que, simultaneamente, eram obesos e residiam em moradia não própria e de 27,9% nos 43 policiais que, simultaneamente, eram obesos e tinham jornada de trabalho maior que oito horas por dia ( Tabela 4 ).

**Tabela 3:** Prevalência de baixa capacidade para o trabalho (CT) segundo combinações dos subestratos de jornada de trabalho, moradia e obesidade em 329 policiais militares. Salvador, BA, 2014.

Jornada > 8 h/dia	Moradia não própria*	Obeso	N	Prevalência de baixa CT	

				n	%
Sim	Sim	Sim	26	9	31,0
Sim	Não	Sim	17	3	17,6
Sim	Sim	Não	148	17	11,5
Sim	Não	Não	71	3	4,2
Não	Sim	Sim	3	0	0,0
Não	Não	Sim	1	0	0,0
Não	Sim	Não	44	1	2,3
Não	Não	Não	19	1	5,3
-	-	-	329	34	10,3

* Alugada, financiada ou outras situações

**Tabela 4:** Prevalência de baixa capacidade para o trabalho (CT) segundo combinações dos subestratos de jornada de trabalho, moradia e obesidade, tomados dois a dois, em 329 policiais militares. Salvador, BA, 2014.

Jornada > 8 h/dia	Moradia não própria*	Obeso	N	Prevalência de baixa CT	

				n	%
-	Sim	Sim	29	9	31,0
-	Sim	Sim	192	18	9,4
-	Não	Não	18	3	16,7
-	Não	Não	90	4	4,4
-	-	-	329	34	10,3
Sim	-	Sim	43	12	27,9
Sim	-	Sim	4	0	0,0
Não	-	Não	219	20	9,1
Não	-	Não	63	2	3,2
-	-	-	329	34	10,3
Sim	Sim	-	174	26	14,9
Sim	Sim	-	47	1	2,1
Não	Não	-	88	6	6,8
Não	Não	-	20	1	5,0
-	-	-	329	34	10,3

* Alugada, financiada ou outras situações

## DISCUSSÃO

Os 329 policiais militares de Salvador apresentaram escore médio do ICT de 37,8 pontos, o que colocaria esta população na categoria de boa CT. Dentre esses 329 policiais, os 95 (28,9%) com ICT moderado deveriam ter a sua capacidade para o trabalho melhorada, e os 34 (10,3%) com ICT baixo deveriam ter a sua capacidade para o trabalho restaurada, segundo a classificação proposta pelos criadores do instrumento^23^ .

A capacidade para o trabalho dos policiais militares de Salvador foi semelhante à relatada para 98 policiais militares (86 do sexo masculino) de Uberaba, em Minas Gerais: 22,4% dos trabalhadores com capacidade ótima, 37,8% com capacidade boa, 29,6% com capacidade moderada e 10,2% com capacidade baixa^3^ . Tal parâmetro também foi avaliado em 42 policiais militares do Batalhão de Operações Especiais de Santa Maria, no Rio Grande do Sul. Esse batalhão é especialmente preparado para atuar em situações especiais como assaltos a banco, sequestros e tumultos generalizados, e seus componentes devem apresentar excelente condicionamento físico e mental. De fato, os policiais do Batalhão de Operações Especiais de Santa Maria caracterizaram-se por elevada proporção de indivíduos com capacidade para o trabalho ótima (40,5%) e boa (47,6%) e menor proporção nos estratos de CT moderada (11,9%) e baixa (0,0%)^12^ . Em 94 policiais finlandeses, o ICT médio foi de 39 pontos, baixo em 7% da amostra, moderado em 21%, bom em 57% e ótimo em 72% da amostra^2^ . Essas proporções revelam melhor capacidade para o trabalho do que as encontradas em policiais de Salvador, com a ressalva de que a média de idade dos policiais finlandeses era bem maior (49 anos, variando de 42 a 61) do que a dos soteropolitanos (36,5 anos, variando de 25 a 54).

Alguns estudos avaliaram a capacidade de trabalho em trabalhadores de outros grupos ocupacionais. Um estudo com 30 bombeiros militares de um município do interior paulista não encontrou indivíduos no estrato de baixa capacidade de trabalho; os demais foram classificados nos estratos de capacidade de trabalho ótima (53,3%), boa (36,7%) e moderada (10,0%)^13^ .

Em resumo, considerando os resultados dos cinco estudos citados anteriormente, a os policiais militares de Salvador caracterizam-se por apresentar elevada proporção de indivíduos nos estratos de capacidade baixa e moderada, em proporções comparáveis apenas às dos policiais militares de Uberaba^3^ . O subgrupo com baixa capacidade para o trabalho caracterizou-se por elevada proporção de policiais que cumpriam jornada diária de trabalho superior a oito horas, residiam em moradia não própria e eram obesos.

Longas jornadas de trabalho já foram associadas à diminuição da capacidade para o trabalho, devido ao estresse decorrente do esforço físico, em trabalhadores metalúrgicos e do comércio varejista^20^ . O esforço físico é um fator de risco bem-estabelecido para a capacidade para o trabalho^21^ .

Desconhecemos estudos que relatassem a associação entre baixa capacidade para o trabalho e a posse da moradia. Residir em moradia não própria (alugada, financiada ou outras formas de posse do imóvel) deve ser tomado como um *proxy* da condição social do policial militar. Nesse caso, residir em casa não própria seria um elo na associação entre baixa condição social e baixa capacidade para o trabalho.

Nossos resultados revelaram a importância da obesidade para a baixa capacidade de trabalho dos policiais. A prevalência da obesidade foi muito maior em policiais militares com baixa CT (25,5%) do que nos policiais com moderada, boa e ótima CT (7,8%). Além disso, a análise das combinações dos substratos de moradia, jornada de trabalho e obesidade revelaram o quanto a última variável contribuiu para a prevalência da baixa CT dos policiais investigados. O excesso de peso representa um problema relevante para a capacidade para o trabalho desses policiais, cuja atividade de policiamento ostensivo em ambientes públicos costuma exigir grande demanda física. A obesidade associa-se a riscos aumentados de doença coronariana do coração, hipertensão arterial, acidente vascular cerebral, certos tipos de câncer, diabetes, litíase biliar, dislipidemia, osteoartrite, gota, doenças pulmonares e apneia do sono, além do sofrimento com o preconceito e discriminação^22^ . Pesquisas sobre qualidade de vida mostram que a obesidade está mais associada à piora dos aspectos físicos que emocionais e que o aumento do peso corporal está diretamente relacionado com piora da capacidade física, da vitalidade e de dores corporais^23^ .

A natureza das exposições e do desfecho que foram objetos desta pesquisa descritiva, de desenho epidemiológico transversal, não nos permite ter certeza quanto ao atendimento de pressupostos para a investigação de causalidade, tais como: a) que a exposição tenha precedido ao desfecho na linha do tempo; b) inexistência de causalidade reversa; c) existência de uma população estável ao longo de um determinado período de tempo; e d) a duração média do desfecho deve ser a mesma, independentemente do grupo de exposição^24 , 25^ . Além disso, não pode-se afastar a importância do “efeito do trabalhador sadio”^26^ , que pode ter agido de forma mais relevante na composição deste grupo de policiais militares, pelo menos de duas formas: a) à entrada na corporação, ao selecionar indivíduos com características diferenciadas para compor os subgrupos de exposição e desfecho e b) ao longo do tempo na corporação, ao excluir aqueles policiais que vinham apresentando baixa capacidade para o trabalho, por motivos diversos (efeito sobrevivência do trabalhador sadio). Uma importante limitação desta pesquisa foi a ausência de algum tipo de avaliação da organização do trabalho, carga de trabalho e cargas psicossociais do trabalho. Às limitações inerentes a um estudo de corte transversal somou-se a dificuldade de trabalhar com uma população vulnerável. Foi necessária a não identificação dos respondentes, visando minimizar as influências das relações de hierarquia características das organizações militares. Antes, durante e após a coleta de dados, foi realizada ampla divulgação na corporação para sensibilizar os policiais a participar do estudo, que possibilita a implantação de um programa voltado à melhoria de sua qualidade de vida relacionada à saúde. Além dos aspectos citados, a realização da coleta por uma única profissional de saúde, pertencente à corporação, possibilitou a incomum proporção de recusas formais encontrada neste estudo (0,0%). Um ponto forte deste estudo foi a utilização de uma amostra selecionada de forma aleatória, representativa dos policiais militares de Salvador, de tamanho relativamente grande.

## CONCLUSÕES

Um em cada 10 policiais militares de Salvador apresenta baixa capacidade para o trabalho, o que pode dificultar ou comprometer o desempenho de suas atividades de policiamento ostensivo. Esse subgrupo de profissionais caracteriza-se por elevada proporção de indivíduos que residiam em moradia não própria, trabalhavam mais de oito horas por dia e, principalmente, eram obesos.

## Footnotes

a Dean AG, Sullivan KM, Soe MM. OpenEpi: Open Source Epidemiologic Statistics for Public Health, www.OpenEpi.com
